# Assessment of a self-assembling peptide gel, SPG-178, in providing a clear operative field for trabeculectomy surgery for glaucoma in an animal model

**DOI:** 10.1038/s41598-020-68171-3

**Published:** 2020-07-09

**Authors:** Kenji Matsushita, Rumi Kawashima, Koji Uesugi, Haruka Okada, Hirokazu Sakaguchi, Andrew J. Quantock, Kohji Nishida

**Affiliations:** 1 Department of Ophthalmology, Osaka University Graduate School of Medicine, 2-2, Yamada-oka, Suita, Osaka 565-0871 Japan; 2 Menicon Co., Ltd., 5-1-10 Takamoridai, Kasugai, Aichi 487-0032 Japan; 3 Department of Advanced Device Medicine, Osaka University Graduate School of Medicine, 2-2, Yamada-oka, Suita, Osaka 565-0871 Japan; 4 School of Optometry and Vision Sciences, Cardiff University, Maindy Road, Cardiff, CF24 4HQ Wales UK; 5 Integrated Frontier Research for Medical Science Division, Institute for Open and Transdisciplinary Research Initiatives, Osaka University, Osaka, Japan

**Keywords:** Medical research, Engineering, Nanoscience and technology

## Abstract

The presence of blood during ophthalmic surgery is problematic, as it can obstruct a surgeon’s view of the operative field. This is particularly true when performing trabeculectomy surgery to enhance ocular fluid outflow and reduce intraocular pressure as a treatment for glaucoma, one of the most common vision loss conditions worldwide. In this study, we investigated the performance of a transparent, self-assembling peptide gel (SPG-178) and its ability to maintain visibility during trabeculectomy surgery. Unlike the hyaluronic acid gel commonly used in ophthalmic surgery, SPG-178 did not permit the ingress of blood into the gel itself. Rather, it forced blood to flow peripherally to the gel. Moreover, if bleeding occurred under the SPG-178 gel, perfusion with saline was able to effectively flush the blood away along the interface between the SPG-178 and the ocular tissue (in this case scleral) to clear the surgical field of view. In experimental trabeculectomy surgeries with mitomycin C used as an adjuvant, there were no differences in the postoperative recovery of intraocular pressure or bleb morphology with or without the use of SPG-178. SPG-178, therefore, when used in a gel formulation, represents a new material for use in intraocular surgery to ensure a clear operative field with likely beneficial treatment outcomes.

## Introduction

The control of bleeding during ophthalmic surgery is key if we are to optimize intraoperative visibility and enhance the surgical outcome^1,2^. To help achieve this, procedures such as heat coagulation and ligation have been performed, and the development of new hemostatic agents and devices is being investigated^3–6^. Ophthalmic surgery is particularly susceptible to intraoperative bleeding because there are many arterioles present in the eye, and attempts at achieving hemostasis have met with limited success. Techniques such as thermo-coagulation, for example, invariably result in the constriction of ocular tissues—especially the conjunctiva and sclera—which can have a detrimental impact on the surgery^7,8^.

Glaucoma is a major reason for irreversible vision loss. Indeed, it is currently ranked as a top cause of blindness worldwide, with its prevalence being closely monitored by the World Health Organization^9,10^. In cases of glaucoma in which drug treatment is inappropriate owing to side effects of the medication, or in patients who experience an insufficient reduction of intraocular pressure (IOP), intraocular surgery can be performed to reduce the IOP^8^. Prior to glaucoma surgery patients often receive IOP-lowering eye-drops. This has unwanted consequences, however, because the eye drops are vasoactive and dilate ocular surface blood vessels to exacerbate the likelihood of bleeding. Good intraoperative visibility is an important issue for glaucoma surgery because it may detrimentally affect the surgical outcome^8^. Here, we investigate the use of a self-assembling peptide gel (SPG-178) as a novel material, which safeguards visibility during glaucoma surgery without the need for heat coagulation and the deleterious effects that accompany this.

**Figure 1:**
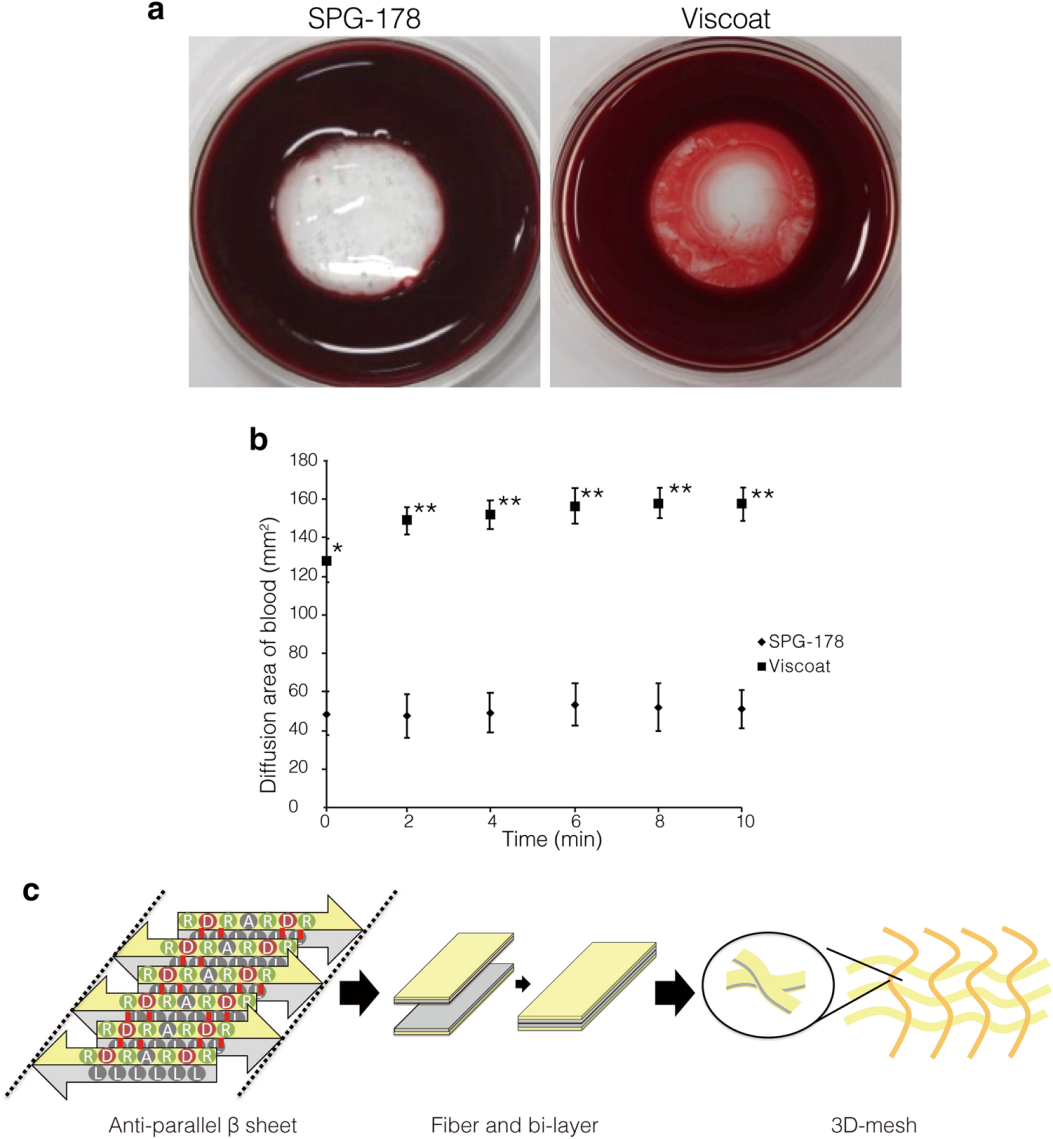
The ingress of blood into the SPG-178 gel. (**a**) The ability of SPG-178 and Viscoat hyaluronic acid to allow the inward diffusion of blood after immersion for 2 min in 2 ml of blood. (**b**) Area occupied by blood in SPG-178 (black diamond) and Viscoat (black square) (p = 0.002 (repeated ANOVA), *p = 0.01 (0 min); **p < 0.01 (2, 4, 6, 8, 10 min) by unpaired Student’s *t* test). The error bars represent the standard deviation of the means (SDs, n = 3). (**c**) The peptide forms an antiparallel beta sheet structure in an aqueous solution by electrostatic interaction and forms one fiber.

The SPG-178 is a peptide consisting of 13 amino acid residues with the sequence RLDLRLALRLDLR, where R, L, D, and A refer to arginine, leucine, aspartic acid, and alanine, respectively^11^. The peptide forms an antiparallel beta sheet structure in aqueous solution via electrostatic interactions (Fig. 1) and a gel, SPG-178, by creating a three-dimensional network of fibers. SPG-178 is neutral and transparent, and when it comes into contact with salt or a bodily fluid such as blood, it forms a boundary^4^ as it hardens^11^. These characteristics have been studied in various medical fields^12–15^. Taking advantage of the unique properties of the SPG-178, herein we report its use to ensure intraoperative visibility applicable to a wide range of ophthalmic operations, not least of which is surgery for glaucoma that, as mentioned, is prone to excessive bleeding.

## Results

### The SPG-178 prohibits the ingress of blood

The diffusion of blood into Viscoat, a hyaluronic acid gel commonly used for ophthalmic surgery, and into the SPG-178 gel was measured and found to be significantly suppressed in the SPG-178 case when compared to that of Viscoat (p = 0.002 (repeated ANOVA), p = 0.01 (0 min), p < 0.01 (2, 4, 6, 8, and 10 min after the immersion of each material in 2 ml of blood) (unpaired Student’s *t* test)) (Fig. 1).

### The SPG-178 excludes blood and maintains visibility of the adjacent tissue in vivo

**Figure 2:**
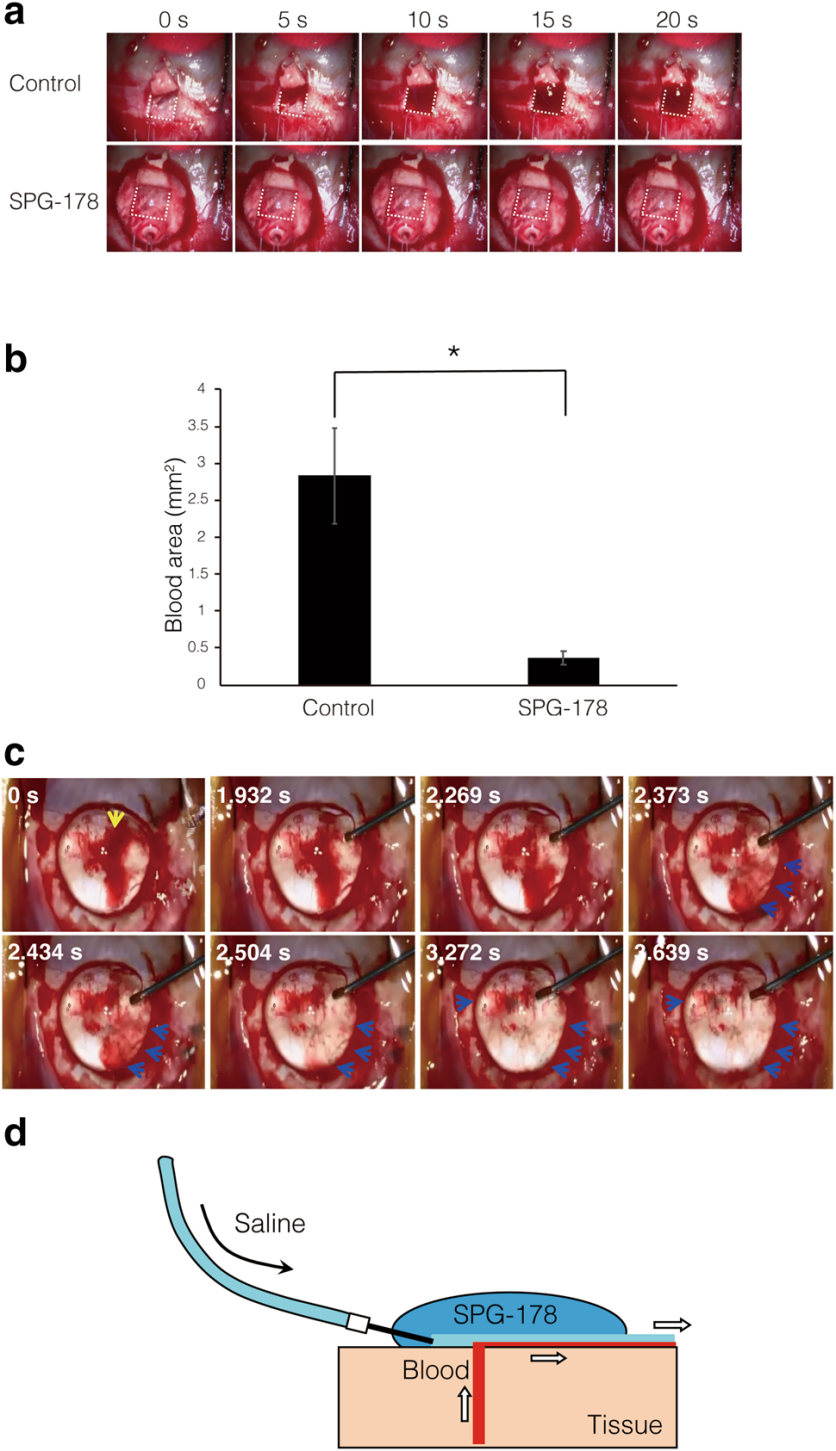
Visibility of the operative field adjacent to SPG-178. (**a**) Visibility of the scleral bed (white dotted line) at each time point with/without SPG-178. (**b**) The area of the scleral bed occupied by blood 30 s after surgery with/without SPG-178 (*p < 0.05 by Mann–Whitney *U*’s test). The error bars represent the SDs (n = 4). (**c**) Representative image of perfusion insertion, with the yellow arrow indicating new bleeding that occurred under the SPG-178 gel. Perfusion insertion with a needle placed between SPG-178 and the sclera resulted in clear visibility under the gel. Blue arrows indicate blood discharged outside the SPG-178 gel. (**d**) Schematic of the dispersal of blood from under SPG-178 via the flow of saline between the gel and the sclera.

To evaluate the ability of the SPG-178 gel to maintain the visibility in an eye wound in vivo, partial-depth, flap-shaped scleral incisions (scleral flaps) were created in the eyes of buphthalmos rabbits. When SPG-178 was applied to the sclera, it excluded the blood and secured the visibility of the operative field within 30 s (Fig. 2a). At this time-point, the area occupied by blood on the sclera was significantly less in the SPG-178 application group compared to the non-application group (p < 0.05, [Mann–Whitney *U* test]) (Fig. 2b). Furthermore, when new bleeding occurred under the SPG-178 gel created by an incision through the gel, saline perfusion was able to flush the blood away from the operative field within a few seconds, along an exit-route formed between the interface of the SPG-178 gel and the scleral bed, which was then clearly visible (Fig. 2c,d).

### SPG-178 and its use for experimental trabeculectomy

**Figure 3:**
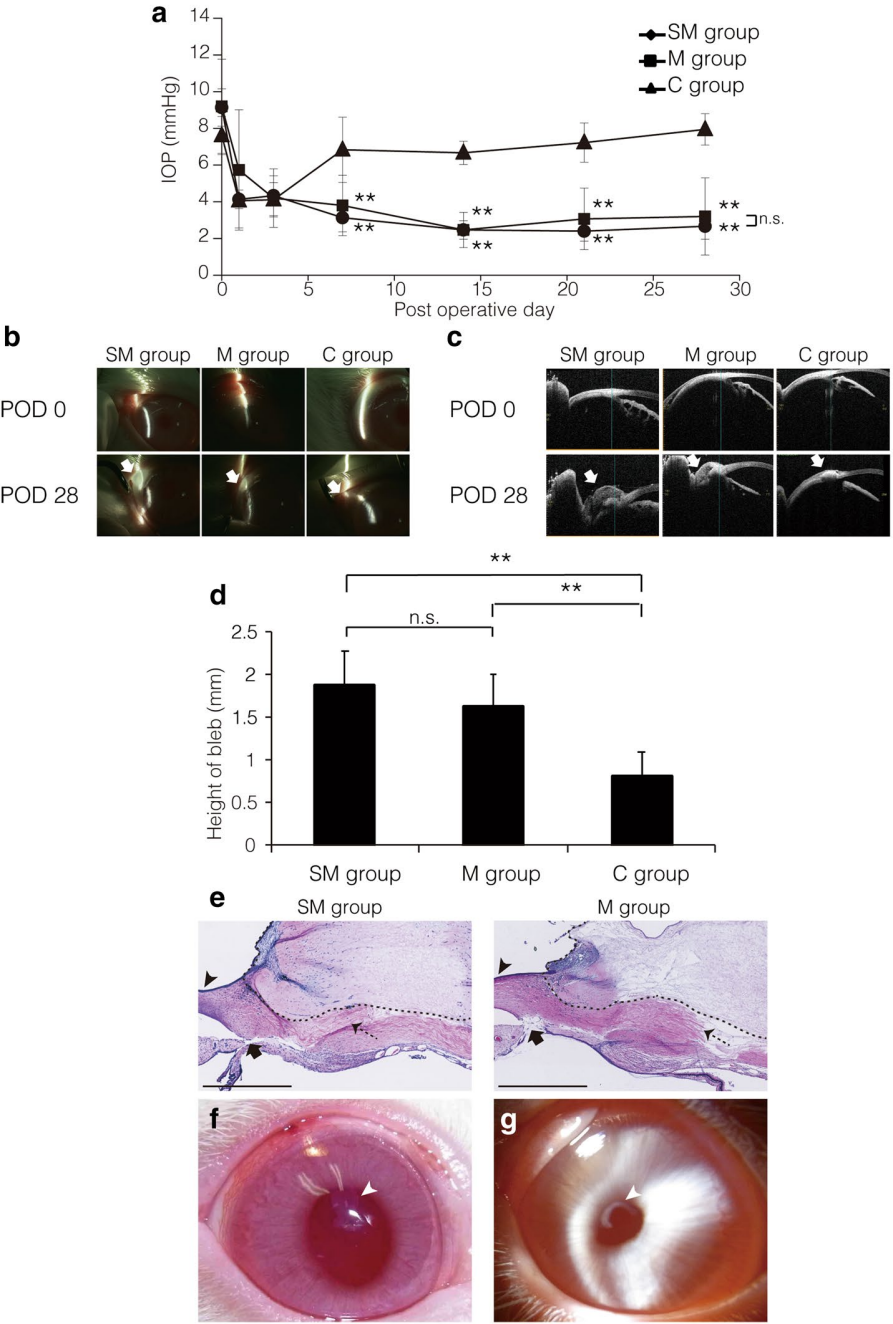
Experimental trabeculectomy with SPG-178. (**a**) Time course of intraocular pressure (IOP) change after a trabeculectomy operation in the SM group (black circles), M group (black squares), and control C group (black triangles). The IOP was significantly lower in the SM and M groups compared to IOP in the C group between 7 and 28 days (p < 0.001 by ANOVA, **p < 0.01; and n.s., not significant by Dunnett’s test). (**b**, **c**) A bleb (white arrow) was formed at postoperative day 28 in the SM, M, and C groups as seen by both slit-lamp observation (**b**) and optical coherence tomography (**c**). (**d**) The height of the bleb at postoperative day 28 was significantly greater in the SM and M groups compared to the C group (p = 0.004 by ANOVA, **p < 0.01; and n.s., not significant by Dunnett’s test). Error bars represent SDs [n = 6 (C group), n = 5 (M and SM groups)]. (**e**) Histology of the sclera on postoperative day 28 in the SM and M groups. The arrowhead indicates the cornea, the arrow the iridectomy wound, the dotted arrow the scleral flap, and the dotted line the bleb. Scale bar = 1 mm. (**f**, **g**) Slit lamp observation after injection of SPG-178 into the anterior chamber. The SPG-178 became cloudy, but the cornea remained clear. (**f**) 4 h after its insertion, (**g**) 2 weeks after the operation. The white arrowhead indicates cloudy SPG-178 adhered to the corneal endothelial surface.

To examine the potential applicability of SPG-178 for glaucoma surgery, 16 rabbits underwent a trabeculectomy performed with SPG-178 and mitomycin C (group SM; n = 5) or with mitomycin C alone (group M; n = 5). A control group (group C) of six rabbits received a trabeculectomy without either mitomycin C or SPG-178. After surgery, IOP was significantly lower in groups SM and M than in control group C throughout the postoperative day 7–28 period [p < 0.001 (ANOVA), p < 0.01 (Dunnett’s test)] (Fig. 3a). The height of the bleb was also significantly larger in groups SM and M than in group C [p = 0.004 (ANOVA), p < 0.01 (Dunnett’s test)] on postoperative day 28 (Fig. 3b–d). No complications were observed in any of the groups, and based on histological analyses no differences were observed between groups SM and M (Fig. 3e), suggesting that, in the short-term at least, SPG-178 is suitable for use in intraocular surgery.

During trabeculectomy surgery, there is a risk of the inadvertent entry of surgical materials into the anterior chamber of the eye, which separates the cornea and lens. To assess the potential effect such an eventuality with SPG-178, small amounts of the gel were inserted into the anterior chamber close to the corneal endothelium and next to the lens using a 26-gauge needle. As a sham control, a 26-gauge needle was briefly placed in contact with the corneal endothelium and the lens. Four hours after its insertion into the anterior chamber, the SPG-178 had become cloudy and visible, and one day later it was seen to be attached to both the corneal endothelium and the anterior surface of the lens (Fig. 3f). The reason for the cloudiness of the SPG-178 in the anterior chamber is believed to be because of an aggregation caused by the electrostatic interaction between charged SPG-178 and sodium, chloride, and protein in the aqueous humor^14^. The SPG-178 that was attached to the surface of the corneal endothelium was turbid and white in colour and kept its shape until five weeks after the operation (Fig. 3g). On the lens, however, the cloudy SPG-178 faded and its outline became unclear one week postoperatively. A week later the gel was barely observable on the lens, and it was not seen at all subsequent to this. In the control sham-operated group, a slight trace of the needle tip placement area was observed the day after surgery, which disappeared by the second week postoperatively. No abnormalities were observed in the anterior segment in the five weeks following surgery.

## Discussion

Materials and techniques to effectively secure intraoperative visibility during eye surgery are sought by the ophthalmic community. Here, we show how an SPG-178 gel not only prohibited the ingress of blood into the body of the gel itself, but also discharged existing blood from areas of the eye to which it was applied. Suppression of blood ingress is due to the fact that a partially impermeable interface is formed between the SPG-178 gel and blood once they come into contact^4^. In contrast, hyaluronic acid gels, which are commonly used in ophthalmic surgery, do not form a barrier to blood at their surface, thus allowing blood to diffuse into the gel. The suppression of blood ingress, accompanied by the high transparency of the gel itself, identifies SPG-178 as a valuable material for use in intraocular surgery ensuring good visibility in the operative field in regions of the eye to which it will be applied.

In this study, the hemostatic effect of the SPG-178 was weak, and when bleeding occurred under the gel, the blood soon dispersed. The rabbits used in this study developed buphthalmos, which meant that they had high blood pressure locally in their eyes. Thus, the hemostatic effect of SPG-178 cannot be fully appreciated by the current experiments, unlike in a previous study^4^ involving rats. Our investigations show that by perfusing saline under the SPG-178 gel, blood that had pooled under the gel could be transferred away without the collapse of the gel (Supplementary Video). The concept behind this is that the action of perfusing saline between SPG-178 and the scleral bed on which it sits results in the formation of an interface between the gel and the sclera which acts as an escape path to flush out the blood (along with the saline, of course) (Fig. 2d). Via this mechanism, fluids can be removed from the operative field and visibility can be maintained even in the presence of new bleeding under the SPG-178.

In trabeculectomy surgery, mitomycin C can be used as an adjuvant to suppress scar formation, maintain blebs and help keep a low IOP^16–19^. The current study reveals no significant differences in maintaining blebs and reducing IOP between experimental surgical groups combining SPG-178 and mitomycin C use, suggesting that SPG-178 elicits no adverse reactions to IOP in the eye following trabeculectomy combined with mitomycin C administration. Experiments were undertaken to examine whether SPG-178 in the scleral bed or the sub-conjunctival space could impair aqueous outflow, or if it could act as a space maintainer to prevent scaring and enhance outflow capacity. This revealed that, in the absence of mitomycin C, operations performed with or without SPG-178 resulted in no difference in IOP (Supplementary Fig. S1), suggesting that SPG-178 neither acts as an enhancer, nor a blocker, of aqueous outflow.

When pieces of SPG-178 were deliberately placed in the anterior chamber, as might happen accidentally during intraocular surgery, they became cloudy and affixed to the anterior surface of the crystalline lens and to the corneal endothelial surface (where they had been placed). Thereafter, the SPG-178 disappeared gradually from the lens surface, most likely washed off by the flow of aqueous humor accompanied by the motion of the iris. SPG-178, on the other hand, persisted longer on the corneal endothelial surface (where there is less flow of aqueous humor), and was still present 5-weeks post-insertion. The persistence of some residual SPG-178 gel in these experiments did not result in any corneal damage or loss of transparency (Fig. 3g). Nevertheless, we cannot rule out the possibility that accidental endothelial contact with large amounts of SPG-178 could conceivably impact upon endothelial function, so the use of SPG-178 may prove to be contraindicated in intraocular interventions or procedures that carry a high risk of perforation.

Here, we provide evidence that SPG-178 is an effective material to maintain a clear intraoperative field during ocular surgery, and is safe, with the caveat that the entry of large amounts of SPG-178 into the anterior chamber is likely to be undesirable and should be avoided. If it were to happen accidentally, the SPG-178 should be washed out with balanced salt solution, and ongoing experiments will ascertain how this is best achieved, with long-term monitoring of the endothelium using specular microscopy to be employed. Thus, the potential value of SPG-178 gel for ophthalmic surgery has been demonstrated using an animal model of trabeculectomy for glaucoma. Our results hold for the short-term, with further validation needed to determine long-term safety. Additionally, we found that if SPG-178 enters the anterior chamber, the surgeon should try to guard against it coming into contact with the corneal endothelium and to implement countermeasures as mentioned above. Here, we focused specifically on experimental trabeculectomy to treat glaucoma, but propose that SPG-178 has potential uses in other eye surgeries (such as those to treat strabismus or to excise a pterygium), and more widely outside the field of ophthalmology for microsurgery in which visibility within the surgical field is important.

## Materials and methods

All experimental procedures adhered to the Association for Research in Vision and Ophthalmology Statement for the Use of Animals in Ophthalmic and Vision Research, and were approved by the Institutional Animal Care and Use Committee and performed according to Animal Experiment guidelines of Osaka University.

### Animal use

Adult New Zealand White rabbits were used in this study. At all times, animals were housed and treated in accordance with the regulations of the Institute of Experimental Animal Sciences, Faculty of Medicine, Osaka University.

### Ingress of blood into SPG-178

To measure the ability of SPG-178 to resist the inward diffusion of blood, 2 ml of blood from a rabbit was placed in a 35-mm disposable petri dish after which 0.5 ml of SPG-178 was immersed within it. Photographs were taken immediately and 2 min after placement of the SPG-178, after which the images were digitized using Image J (NIH)^20^. The area of blood ingress into the SPG-178 was quantitatively analyzed. The hyaluronic acid gel, Viscoat (Alcon, USA), was similarly tested as a comparator material commonly used in ophthalmic surgery.

### The removal of blood under SPG-178

A scleral flap (3 × 3 mm), which is a partial thickness planar incision in the sclera of the eye often used in ophthalmic surgery, was created unilaterally in eight buphthalmos rabbits^21^. This induced bleeding, and in the non-SPG-178 control group of four animals the area occupied by blood in the bed of the scleral flap was analyzed using Image J (NIH) 30 s after the creation of the wound. In the treatment group, SPG-178 was applied to the scleral bed of four eyes immediately after the creation of the scleral flap, and the area occupied by blood 30 s after this application was quantified. SPG-178 was then applied to 5 regions of the sclera of one buphthalmos rabbit after which a scleral incision was created through each. Physiological saline was then perfused under the SPG-178 in an attempt to counteract the bleeding. The time from saline perfusion to regaining visibility of the sclera owing to the removal of blood was recorded.

### Glaucoma filtration surgery model

For the glaucoma filtration surgery model, rabbits were anesthetized with an intramuscular injection of ketamine (15 mg/kg) (Daiichi Sankyo Propharma) and xylazine (10 mg/kg) (Bayer Yakuhin). An eyelid speculum was put in place, and the eye fixed with a corneal traction suture (7–0 Silk, Johnson & Johnson). We then injected xylocaine (0.3–0.5 ml) under the Tenon tissue as an additional local anesthesia, and made a 6-mm fornix-based conjunctival flap with the Tenon’s capsule, 5 mm in length. Next, we made a 3 × 3 mm fornix-based scleral flap and placed 0.04% mitomycin C (Kyowa Kirin) into the conjunctival pocket for 3 min, which was then washed away with 50 ml of saline. Finally, we created a 2 × 1 mm scleral window at the limbus and performed a peripheral iridectomy, followed by a conjunctival suture with 10–0 nylon (MANI) and the injection of 0.5 ml dexamethasone into the subconjunctiva. After surgery, 0.3% ofloxacin ointment (Santen Pharmaceutical) was applied to the eye. Topical administrations of 0.1% betamethasone sodium phosphate (SHIONOGI) and 0.5% levofloxacin hydrate ophthalmic solution (Santen Pharmaceutical) were applied three times a day for three days postoperatively. All surgeries were performed by the same researcher (K.M).

### SPG-178 use in trabeculectomy surgery

We performed a trabeculectomy with the use of SPG-178 peptide gel in a rabbit ocular surgery model as above. Gel (0.3–0.4 g) was injected onto the surface of the anterior sclera so that it covered the entire scleral flap as it was made. The scleral flap was created through the gel, after which it was washed with saline. After confirming that no SPG-178 gel remained under the scleral flap or Tenon’s capsule, we created a scleral window and performed an iridectomy. SPG-178 is a peptide consisting of 13 amino acid residues. The peptide forms an antiparallel beta sheet structure in aqueous solution via electrostatic interactions and a gel, SPG-178, by creating a three-dimensional network of fibers (Fig. 1c)^14^. SPG-178 is neutral and transparent and when it comes into contact with salt or a bodily fluid such as blood, it forms a boundary as it hardens. These properties of the SPG-178 gel mean that we can easily remove it by wiping or washing it out with a fluid such as balanced salt solution.

Sixteen New Zealand White rabbits underwent trabeculectomy unilaterally. Five animals underwent surgery with combined SPG-178 and mitomycin C use (SM group); five received surgery with mitomycin C, but not SPG-178 (M group); and six received a normal trabeculectomy without mitomycin C or SPG-178 (the control, C group). Slit-lamp microscopy, IOP measurements, and ocular coherence tomography were used to examine in each eye immediately after surgery and at postoperative days 1, 3, 7, 14, 21, and 28. After each examination, the eyes were harvested and histological analyses (HE staining) of the trabeculectomy region were performed.

### Examination of SPG-178 in the anterior chamber

In three rabbit eyes, SPG-178 was applied immediately adjacent to the corneal endothelium and lens using a 26-gauge needle. As a sham treatment control, a 26-gauge needle was brought into contact with the endothelium and lens of two rabbit eyes. The eyes were observed by slit-lamp microscopy on postoperative days 1, 3, 7, 14, and 35.

### Statistics

Statistical analyses were performed using JMP Pro 12 software (SAS Institute Inc., Cary, NC, USA).

## Supplementary information

Supplementary file1

Supplementary file2
