# Supplementary material for: Assessment of a self-assembling peptide gel, SPG-178, in providing a clear operative field for trabeculectomy surgery for glaucoma in an animal model
Source: Sci Rep. 2020 Jul 9;10:11326. doi: 10.1038/s41598-020-68171-3 (PMC7347556; doi:10.1038/s41598-020-68171-3)
Supplement: Supplementary file 1 — Supplementary file1 [file 41598_2020_68171_MOESM1_ESM.docx]

**Supplementary Information**

**Assessment of a self-assembling peptide gel, SPG-178, in providing a clear operative field for trabeculectomy surgery for glaucoma in an animal model.**

Kenji Matsushita^1^*, Rumi Kawashima^1^, Koji Uesugi^1, 2^, Haruka Okada^2^, Hirokazu Sakaguchi^3^, Andrew J. Quantock^4^, Kohji Nishida^1,5^*

^1^ Department of Ophthalmology, Osaka University Graduate School of Medicine, 2-2, Yamada-oka, Suita, Osaka 565-0871, Japan

^2^ Menicon Co., Ltd., 5-1-10 Takamoridai, Kasugai, Aichi 487-0032, Japan

^3^ Department of Advanced Device Medicine, Osaka University Graduate School of Medicine, 2-2, Yamada-oka, Suita, Osaka 565-0871, Japan

^4^ School of Optometry and Vision Sciences, Cardiff University, Maindy Road, Cardiff, Wales, CF24 4HQ, United Kingdom

^5^ Integrated Frontier Research for Medical Science Division, Institute for Open and Transdisciplinary Research Initiatives, Osaka University

*Corresponding Authors: [kenmatsu@ophthal.med.osaka-u.ac.jp](mailto:kenmatsu@ophthal.med.osaka-u.ac.jp) and knishida@ophthal.med.osaka-u.ac.jp

**Supplementary Methods**

We performed trabeculectomy surgery without mitomycin C or SPG-178, in three eyes of three rabbits. SPG-178 was placed into the bleb after trabeculectomy (S group) to examine if gel that remained in the scleral bed or the sub-conjunctival space could impair aqueous outflow, or if it acted as a space maintainer to prevent scaring and enhance outflow capacity. IOP was measured at postoperative days 1, 3, 7, 14, 21, and 28.

**Supplementary Figure**

**Figure S1. The effect of SPG-178 on intraocular pressure (IOP).**

**
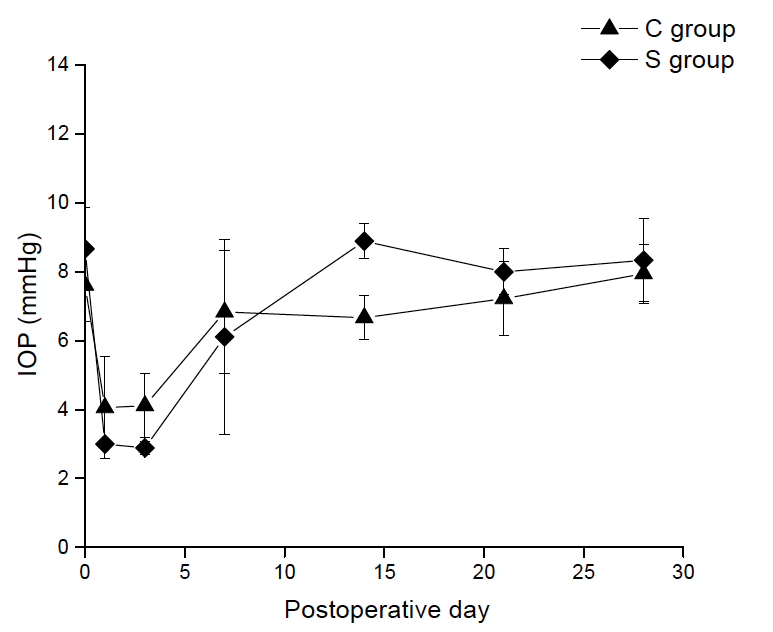
**

Time course of intraocular pressure (IOP) (mean ± SD) after trabeculectomy operations performed without SPG-178 or mitomycin C (n=6, C group, black triangles), and with SPG-178 but not mitomycin C (n=3, S group, black rhombus). There was no statistical difference (p>0.05) in IOP between the two groups at any postoperative time point.

**Supplementary Video**

**Representative effect of a perfusion insertion.**

When a new bleed occurred under the SPG-178 gel we made a perfusion insertion (arrowed) between the gel and the sclera. This resulted in clear visibility of the intraoperative field under the gel as blood was discharged away from the SPG-178.
